# In Vitro Evaluation of the Effect of Size and PEGylation on Inhalable Liposomes for Pulmonary Drug Delivery

**DOI:** 10.3390/nano16030200

**Published:** 2026-02-03

**Authors:** Juliana Carrillo-Romero, Laura Fernández-Méndez, Endika de la Iglesia, Alberto Katsumiti, Lorena Germán, Desirè Di Silvio, Jesús Ruíz-Cabello, Susana Carregal-Romero, Felipe Goñi-de-Cerio

**Affiliations:** 1 GAIKER Technology Centre, Basque Research and Technology Alliance (BRTA), 48170 Zamudio, Spain; carrillo@gaiker.es (J.C.-R.); delaiglesia@gaiker.es (E.d.l.I.); katsumiti@gaiker.es (A.K.); lorena.german@gmail.com (L.G.); 2 Center for Cooperative Research in Biomaterials (CIC biomaGUNE), Basque Research and Technology Alliance (BRTA), 20014 San Sebastián, Spain; lfernandez@cicbiomagune.es (L.F.-M.); ddisilvio@cicbiomagune.es (D.D.S.); jruizcabello@cicbiomagune.es (J.R.-C.); scarregal@cicbiomagune.es (S.C.-R.); 3 Faculty of Science and Technology, Euskal Herriko Unibertsitatea (UPV/EHU), 48940 Leioa, Spain; 4 CIBER de Enfermedades Respiratorias (CIBERES), 28029 Madrid, Spain; 5 Ikerbasque, Basque Foundation for Science, 48013 Bilbao, Spain; 6 NMR and Imaging in Biomedicine Group, Department of Chemistry in Pharmaceutical Sciences, Pharmacy School, University Complutense Madrid, 28040 Madrid, Spain

**Keywords:** liposomes, pulmonary drug delivery, PEGylation, mucus penetration, air–liquid interface

## Abstract

The development of effective inhalable drugs remains a key challenge in the treatment of pulmonary diseases, due to the physiological barriers of the respiratory tract and the lack of predictive models that accurately reproduce the human lung environment. In this context, liposomes (LP) have emerged as promising nanocarriers for pulmonary drug delivery due to their high biocompatibility, surfactant-like composition, capacity to encapsulate both hydrophilic and lipophilic drugs, and potential to provide sustained drug release while reducing systemic toxicity. This study evaluates the influence of size and PEGylation on their physicochemical properties, cytotoxicity, interaction with the pulmonary mucus, and cellular internalisation. LP of 100 nm (LP 100), 200 nm (LP 200), and 600 nm (LP 600) were characterised physiochemically and evaluated in pulmonary cell lines (A549 and Calu-3) exposed in liquid–liquid interface (LLI) and air–liquid interface (ALI) by nebulisation. In addition, artificial pulmonary mucus (APM) was employed to analyse LP penetration through the pulmonary mucus barrier. Results indicate that LP 100 exhibits greater colloidal stability, lower cytotoxicity, and sustained migration through the APM over time with respect to larger particles. PEGylation of LP 100 (LP-PEG) further increases their stability and ability to penetrate the APM, although cellular internalisation is reduced due to the steric effect of the PEG coating. These findings highlight the importance of adjusting the size and surface modifications of LPs according to the therapeutic target of the drug, optimising their persistence on the epithelial surface or their cellular uptake.

## 1. Introduction

Respiratory diseases are among the most significant causes of mortality and morbidity worldwide. According to the Forum of International Respiratory Societies (FIRS), the five major lung diseases (chronic obstructive pulmonary disease (COPD), asthma, acute respiratory infections, tuberculosis, and lung cancer) alone affect more than 500 million people [1]. Despite increasing research into the development of treatments to prevent and treat respiratory diseases, several limitations still hinder the development of effective therapies.

Among the different routes of drug administration, inhalation stands out for delivering drugs directly to the lungs, enabling a faster therapeutic onset, lower effective doses, and reduced systemic side effects while bypassing first-pass metabolism and improving bioavailability [2,3,4,5]. However, the respiratory tract contains several barriers and mechanisms to remove foreign particles, including the mucociliary clearance system, which is the primary mechanism of airway protection against inhalation of toxic and infectious compounds [6,7]. It consists of a mucus layer that captures inhaled particles and removes them by cilia-driven forces and a periciliary layer (PCL) that facilitates cell lubrication and optimises ciliary movement [8]. Pulmonary mucus is a complex viscoelastic gel composed mainly of water, glycoproteins, lipids, and mineral salts [9]. Its thickness ranges from 10 to 30 µm in the trachea and 2 to 5 µm in the bronchi under healthy conditions. However, under pathological conditions such as cystic fibrosis or COPD, pulmonary mucus not only increases in thickness but also undergoes significant compositional changes, including higher mucin concentration, increased DNA and protein content, and reduced hydration. These alterations result in a denser and more viscoelastic mucus network, which limits the diffusion and penetration of inhaled drug formulations [10,11,12]. Consequently, understanding how nanocarrier properties influence their interaction with altered pulmonary mucus is crucial for the rational design of effective inhalable drug delivery systems.

To overcome these challenges, liposomes (LPs) have been established as one of the most promising therapeutic strategies for respiratory diseases [13]. LPs consist of a spherical lipid bilayer and an aqueous core with the ability to encapsulate both hydrophilic and lipophilic drugs with a high loading capacity [14,15]. Their phospholipid composition resembles lung surfactant [16], promoting uniform distribution on the pulmonary epithelium, enhancing stability, and optimising drug release while mitigating immune responses and clearance by the mucociliary system or alveolar macrophages. Altogether, LPs confer biocompatibility, biodegradability, and sustained release, reducing systemic toxicity [17,18].

LPs are highly versatile in terms of size, charge, and surface properties, enabling either mucoadhesive or mucopenetrating behaviour [19]. Liposomes with diameters below 200 nm are generally considered more suitable for pulmonary delivery, as they can diffuse through the mucus mesh, reach the lung epithelium, and be internalised by cells, thereby enabling systemic distribution, whereas larger particles (>500 nm) are more likely to be sterically trapped within the mucus and subsequently cleared [7,20]. Steric coating of LPs with polymers such as polyethylene glycol (PEG) increases their stability and prolongs circulation time in the bloodstream by providing steric hindrance that prevents aggregation and mucociliary and immune clearance [21,22,23,24]. At the clinical level, LPs were the first nanomedicine to be commercialised and applied to humans [25]. In the context of respiratory diseases, amikacin liposome inhalation suspension (ARIKAYCE^®^, Insmed Incorporated, Bridgewater, NJ, USA) is the first and only inhalable liposomal drug approved by the FDA for the treatment of respiratory infections caused by *Mycobacterium avium* complex (MAC) [26].

Traditionally, animal models have been used to evaluate pulmonary drug delivery, but differences in the lung architecture and specific immunological and metabolic responses limit their translational relevance [27]. In this regard, in vitro lung models provide physiologically relevant, cost-effective, and reproducible alternatives. In contrast to conventional submerged liquid–liquid interface (LLI) cultures, where cells are fully covered by culture medium, air–liquid interface (ALI) models expose the apical surface of the epithelium to air, more closely mimicking the physiological conditions of the respiratory tract [28]. This configuration promotes epithelial polarisation and differentiation, supports the secretion of a protective layer of mucus and/or lung surfactant, and preserves the physiological transport of growth factors, nutrients, and cell signalling molecules across the epithelium. Moreover, ALI cultures enable direct aerosol deposition onto the cell surface, thereby reproducing inhalation exposure more realistically [29,30,31,32]. In this context, commercial ALI aerosol exposure systems such as the VITROCELL Cloud (VITROCELL^®^ Systems GmbH, Waldkirch, Germany) allow the controlled nebulisation and homogeneous deposition of liquid aerosols, ensuring highly reproducible and well-defined particle delivery under physiologically relevant conditions [33].

Complementary to cellular models, the assessment of drug interactions with lung mucus is essential for understanding the efficacy of therapies targeting this organ. For this purpose, native mucus isolated directly from human or animal lungs provides a realistic representation of physiological conditions. However, the availability of native mucus is limited, and its composition may vary between individuals or species, and it can be altered during collection and storage, affecting the reproducibility of results [34]. In contrast, mucus produced by epithelial cells under specific conditions, as well as artificial pulmonary mucus (APM), which mimics the physicochemical properties of a native mucus and can be consistently reproduced under controlled conditions, represent highly effective alternative models [35].

This study aims to evaluate the influence of LP size and surface properties on their interactions and penetration through cellular barriers and biological fluids in the lung. A comprehensive set of in vitro assays was conducted, as well as physicochemical characterisation of LPs (100 nm, 200 nm, and 600 nm) using dynamic light scattering (DLS), transmission electron microscopy (TEM), and atomic force microscopy (AFM). Additionally, the effect of PEG coating was specifically evaluated in LPs of 100 nm. Cytotoxicity and cellular uptake were assessed in two pulmonary cell lines (A549 and Calu-3) under LLI and ALI conditions using the VITROCELL Cloud system (VITROCELL Systems GmbH, Waldkirch, Germany) for ALI exposure. Complementarily, LP interaction with pulmonary mucus was analysed through rheological studies, penetration assays, and confocal microscopy using APM. This approach provides insights into the behaviour of LPs in the lung environment and may guide the development of more effective inhalable drug delivery systems.

## 2. Materials and Methods

### 2.1. Liposome Synthesis

Liposomes were prepared using a thin-film hydration method followed by extrusion [36]. Briefly, lipid mixtures (150 µmol) were dissolved in a 6:1 (*v*/*v*) mixture of chloroform and methanol. Two different lipid compositions were prepared: the first contained 1,2-dipalmitoyl-sn-glycero-3-phosphocholine (DPPC, molar fraction x = 0.5) and 1,2-dioleoyl-sn-glycero-3-phosphoethanolamine (DOPE, molar fraction x = 0.5); the second contained DPPC (molar fraction x = 0.67), DOPE (molar fraction x = 0.27), and 2-distearoyl-sn-glycero-3-phosphoethanolamine-N-[methoxy(polyethylene glycol)-2000] (DSPE-PEG, molar fraction x = 0.07) (all purchased from Avanti Polar Lipids, Alabaster, AL, USA).

To fluorescently label the liposomes, 33 µL of a 1 mg/mL solution of 1,1′-dioctadecyl-3,3,3′,3′-tetramethylindotricarbocyanine iodide (DiOC18) (Thermo Fisher Scientific, D12731, Waltham, MA, USA) was added to the lipid mixture before lipid film formation. The lipid films were prepared by evaporating chloroform and methanol using a rotary evaporation system under vacuum at 30 °C, followed by drying under a nitrogen stream for 1 h.

The resulting thin lipid film was rehydrated using HPLC-grade water (Thermo Fisher Scientific, W/0106/14, Waltham, MA, USA) to form a liposome suspension. This suspension was then extruded at 45 °C through polycarbonate membrane filters (Whatman PLC, Cytiva, Maidstone, UK) of decreasing pore size: 400 nm (2 times), 200 nm (4 times), and 100 nm (8 times). After extrusion, the liposomes were washed through a 30 kDa molecular weight cut-off (MWCO) filter (Sigma-Aldrich, PLTK06210, St. Louis, MO, USA) at 4500 rpm.

Three different sizes of DPPC:DOPE liposomes were synthesised: one lipid mixture was extruded through the 400, 200, and 100 nm filters to obtain liposomes of 100 nm (LP 100 nm); a second mixture was extruded through the 400 and 200 nm filters to obtain liposomes of 200 nm (LP 200 nm); and the third mixture was extruded through the 400 nm filter only to obtain liposomes of 600 nm (LP 600 nm). Additionally, LP 100 nm were functionalised with 7% DSPE-PEG (LP-PEG).

### 2.2. Liposome Characterisation

#### 2.2.1. Lipid Content

The total lipid concentration of the liposome formulations was quantified according to the method described by Rouser et al. [37]. A calibration curve was first established by adding 0, 40, 60, 80, 100, 120, and 160 μL of 0.5 M phosphate solution (sodium phosphate monobasic, NaH_2_PO_4_-2H_2_O, Sigma-Aldrich, 04269, St. Louis, MO, USA) to glass test tubes. Aliquots (5–10 μL) of liposome suspensions with an estimated phospholipid content between 10 and 80 nmol were added to separate test tubes. The standards and liposome samples were digested at 180 °C in a dry bath block until complete evaporation.

After evaporation and cooling, 0.3 mL of perchloric acid (Sigma-Aldrich, 244252, St. Louis, MO, USA) was added to each tube, and the samples were reheated at 180 °C for 45 min. To prevent acid evaporation, the tubes were covered with glass beads, which promoted condensation and reduced the volume loss. After cooling, 1 mL of ultrapure water, 0.5 mL of molybdate solution (Sigma-Aldrich, 69888, St. Louis, MO, USA), and 0.5 mL of freshly prepared ascorbic acid solution (Sigma-Aldrich, V-038, St. Louis, MO, USA) were added to each tube. The contents were mixed, and the tubes were then heated at 100 °C for 5 min.

Finally, the absorbance of both the standard and liposome samples was measured using UV–Vis spectroscopy (Varian Cary 5000 UV–Vis–NIR Spectrophotometer, Agilent Technologies, Santa Clara, CA, USA) at 797 nm. The phosphate content of the liposome formulations was determined by comparing the absorbance values with the calibration curve corresponding to phosphate concentrations of 0, 20, 30, 40, 50, 60, and 80 nmol.

#### 2.2.2. Particle Size, Polydispersity, and ζ-Potential Measurements

The hydrodynamic diameter (d_h_), polydispersity index (PDI), and ζ-potential of the liposome samples were measured using a Zetasizer NanoZS (Malvern, Worcestershire, UK). For size analysis, samples were diluted to a concentration of 0.6 μmol/mL phospholipids in HPLC-grade water. The ζ-potential was determined at the same concentration used for size analysis.

#### 2.2.3. Cryo-TEM Microscopy

TEM grids of liposomes were prepared using Vitrobot, with 1 μL of sample applied to glow-discharged ultrathin carbon film grids (Ted Pella, Inc., 01824, Redding, CA, USA). The grids were prepared at 4 °C and 100% humidity before being fully immersed in ethane. Imaging was performed using a JEOL JEM 2100F transmission electron microscope (JEOL Ltd., Tokyo, Japan) operating at 120 kV with a cryo-transfer specimen holder (Gatan Model 626, Gatan, Pleasanton, CA, USA).

#### 2.2.4. Atomic Force Microscopy (AFM)

AFM imaging was performed using a Veeco Multimode 8-HR microscope (Bruker, Santa Barbara, CA, USA) working with a liquid cell. Samples were prepared following the protocol of Takechi-Haraya et al. [38] by coating glass coverslips with a BSA solution. Experiments were performed in Peak Force Tapping mode using a Scanasyst-Fluid probe (Bruker, Santa Barbara, CA, USA) with K = 0.7 N/m and f = 150 kHz. Images were analysed using the Nanoscope Analysis 2.0 software (Bruker).

### 2.3. Cell Culture

Calu-3 (HTB-55), derived from human bronchial epithelial cells, and A549 (CCL-185), derived from human alveolar epithelial cells, were obtained from the American Type Culture Collection (ATCC, Manassas, VA, USA). Both cell lines were cultured in minimum essential medium (MEM; Sigma-Aldrich, M4655, St. Louis, MO, USA) + 10% foetal bovine serum (FBS, Gibco, 11563397, Thermo Fisher Scientific, Waltham, MA, USA) and 1% penicillin–streptomycin (P/S) solution (Sigma-Aldrich, P4083, St. Louis, MO, USA). The cells were maintained at 37 °C and 5% CO_2_, and the culture medium was replaced every 2–3 days until they reached confluency.

The Calu-3 cell line (HTB-55) and the A549 cell line (CCL-185) were obtained from the American Type Culture Collection (ATCC, Manassas, VA, USA). Calu-3 cells are derived from human bronchial epithelium and are capable of forming tight junctions and producing mucus under air–liquid interface conditions, whereas A549 cells originate from human alveolar epithelial cells and are commonly used as a model of the distal lung epithelium.

### 2.4. Cell Viability Assay

Cell lines were exposed for 24 h to 1:2 dilutions of LP of different sizes (100 nm, 200 nm, and 600 nm) and LP-PEG starting at a concentration of 2 mg/mL in MEM + 10% FBS to determine their cytotoxic effect. After exposure, cells were washed with phosphate-buffered saline (PBS), and cell viability was assessed by incubating the cells with MTT reagent (0.4 mg/mL, Sigma-Aldrich, M2003-1G, St. Louis, MO, USA) at 37 °C and 5% CO_2_ for 2 h. The culture medium was then removed, and 100 µL/well of DMSO (PanReac AppliChem, A3672, Barcelona, Spain) was added to dissolve the formed formazan crystals. The plate was kept under constant agitation and darkness for 15 min at room temperature to obtain a homogeneous solution. Absorbance was measured at 540 nm using a spectrophotometer reader (Varioskan™ Lux, Thermo Fisher Scientific, Waltham, MA, USA). Cell viability was calculated as the percentage of viability with respect to non-treated cells in the culture medium (negative control). CdSO_4_ (Sigma-Aldrich, 383082, St. Louis, MO, USA) was used as a positive control to validate the experimental setup and methodology. 

### 2.5. Cellular Uptake of LP 

#### 2.5.1. Liquid-Liquid Interface Exposure

For LLI assays, the A549 cell line was cultured at a density of 2 × 10^5^ cell/mL in 24-well plates and incubated for 24 h to allow confluency. Calu-3 cell line was cultured in 24-well polyethylene terephthalate (PET) inserts with a pore size of 0.4 µm, a diameter of 6.5 mm, and a growth area of 0.33 cm^2^ (Transwell^®^, Corning™, 3470, Kennebunk, ME, USA) for 2 days in submerged conditions at 5 × 10^5^ cell/mL. Subsequently, the apical medium was removed, and the cells were exposed to air for 19 days on the apical side while remaining in contact with the culture medium on the basolateral side. For the Calu-3 cell line, once a week until the experiment, culture medium was added to the apical compartment, and the trans-epithelial electrical resistance (TEER) was monitored. The apical medium was then removed. TEER values ≥ 300 Ω-cm^2^ indicated a tight monolayer. Then, for both cell types, the culture medium was replaced with fresh culture medium with LPs of three different sizes (100 nm, 200 nm, and 600 nm) and LP-PEG at three different concentrations (0.1, 0.5, and 1 mg/mL) and six different time points (0.25 h, 1 h, 2 h, 4 h, 6 h, and 16 h). After removal of the treatment, both cell lines were washed with PBS and trypsinised with 0.25% (*w*/*v*) trypsin-0.53 mM EDTA (Gibco™, 11560626, Thermo Fisher Scientific, Waltham, MA, USA) to assess cell uptake by flow cytometry (Beckman Coulter, Brea, CA, USA).

#### 2.5.2. Air–Liquid Interface Exposure

For ALI assays, A549 and Calu-3 cell lines were cultured in 24-well PET Transwell inserts (0.4 µm pore size, 6.5 mm diameter, and 0.33 cm^2^ growth area). The A549 cell line was cultured at 2.5 × 10^5^ cell/mL in LLI for 24 h and then exposed to ALI for 48 h prior to the experiment. The Calu-3 cell line was cultured under the same conditions as mentioned for submerged exposure. Subsequently, for both cell lines, inserts were placed in the VITROCELL Cloud Exposure System. The equipment was developed specifically for ALI exposure assays and is composed of a 12-well chamber (8 for exposure, 1 integrated Quartz Crystal Microbalance (QCM), and 3 for control) connected to a heating block to maintain a constant temperature of 37 °C and a nebuliser (Aeroneb Lab^®^, Kent Scientific, Torrington, CT, USA) on top of the chamber. This device produces an aerosolised cloud of nanoparticles that precipitates uniformly onto the cells. Then, both cell lines were nebulised with 200 µL of LPs of different sizes (100 nm, 200 nm, and 600 nm) and LP-PEG in PBS for 15 min to obtain uniform deposition on the cell surface. For the A549 cell line, two concentrations (0.1 mg/mL and 0.5 mg/mL) and six different time points (0.25 h, 1 h, 2 h, 4 h, 6 h and 16 h) were evaluated, and in the case of the Calu-3 cell line, two different time points (6 h and 16 h) and one concentration (0.5 mg/mL) were evaluated. In the ALI assays, the concentration of 1 mg/mL was not included in the study due to the potential risk of nebuliser clogging at concentrations above 0.5 mg/mL, which was observed during preliminary testing. After each exposure, the inserts were placed in a 24-well plate at 37 °C and 5% CO_2_ for an estimated time period, with the culture medium on the basolateral side of the insert. The cells were collected for exposure to LLI.

### 2.6. Synthesis of Artificial Pulmonary Mucus (APM)

A standardised procedure was followed for the synthesis of artificial pulmonary mucus [39]. Briefly, 8 mL of MilliQ water was mixed with 50 mg of NaCl (Sigma-Aldrich, S9888, St. Louis, MO, USA), 22 mg of KCl (Sigma-Aldrich, 58221, St. Louis, MO, USA), 50 mg of pig stomach mucin type II (Sigma-Aldrich, M2378, St. Louis, MO, USA), 18.1 mg of Tris ≥ 99.8% (Bio-Rad Laboratories, 1610716, Hercules, CA, USA), 50 mg of low-molecular-weight deoxyribonucleic acid from salmon sperm (Sigma-Aldrich, 31149, St. Louis, MO, USA), and 100 µL of 0.15M diethylene triamine pentaacetic acid (DTPA; Sigma-Aldrich, D6518, St. Louis, MO, USA). The mixture was stirred continuously at 800 rpm for 24 h. Subsequently, 90 mg of poly(acrylic) acid (PAA) (Lubrizol, Carbopol^®^ 974P NF Polymer, Wickliffe, OH, USA) and 50 mg of Casamino Acids (OmniPur^®^, Calbiochem^®^; Sigma-Aldrich, 2240-OP, St. Louis, MO, USA) were added. The mixture was stirred again for 24 h. Tris 1 M was then added to the solution to adjust the pH to 7.0. The final volume was adjusted to 10 mL, and experiments were conducted immediately after preparation.

### 2.7. Rheology

Rheological behaviour of mucus samples was studied by an oscillatory test using a rotational rheometer from ANTON PAAR MCR 501 (Anton Paar GmbH, Graz, Austria) with plate–plate geometry (plate diameter of 50 mm). All measurements were performed at 23 °C. Amplitude sweeps from 0.1 to 10% strain at 6.283 rad/s (1 Hz) were performed to determine the linear viscoelastic region. Then, the elastic/storage (G′), viscous/loss (G″) modulus, and complex viscosity (η*) were analysed by sweep frequency experiments in a range of 0.1–100 rad/s at 0.5% strain. APM sample mixed with PBS was used as the negative control (C−).

### 2.8. In Vitro Penetration Assay Across APM

The ability of LPs of different sizes (100, 200, and 600 nm) and LP-PEG to penetrate the pulmonary mucus was evaluated across the interaction with APM. The assay was carried out in PET inserts with a pore size of 1 µm, a diameter of 6.4 mm, and a growth area of 0.3 cm^2^ (Falcon^®^, Corning™, 353104, Kennebunk, ME, USA). A total of 200 µL of PBS 1X was added to the acceptor chambers of the inserts, and 100 µL of APM was placed over the donor chambers. To remove all bubbles and generate a uniform mucus layer, the plate was continuously shaken at 37 °C for 10 min at 200 rpm on a shaking board (Heidolph Instruments, Schwabach, Germany). Then, the mucus layer was covered with 50 µL of the sample (1 mg/mL) dispersed in PBS 1×, and the plate was incubated at 37 °C at six different time points (1 h, 2 h, 4 h, 6 h, 16 h, and 24 h). Following the collection of apical and basolateral samples, fluorescence intensity (excitation wavelength: 480 nm; emission wavelength: 510 nm) was evaluated by spectrofluorimetric measurements using a microplate reader (Varioskan Lux, ThermoFisher Scientific, Waltham, MA, USA). Transwell inserts with APM and without LP were used as negative controls.

### 2.9. Confocal Microscopy

The diffusion of dye-labelled LPs was evaluated in the APM stained with a staining kit (MemBrite^®^ Fix 660/680, Biotium, 30098-T, Fremont, CA, USA) at 0.5×. A total of 150 µL/well of the APM was deposited on a µ-Slide 8 Well^high^ (Ibidi GmbH, Gräfelfing, Germany) and horizontally shaken to form a layer with a uniform thickness. Then, 4 µL of the compound (1 mg/mL) was applied to the surface of the mucus and incubated for 4 h at 37 °C. The sample was then transferred to a confocal laser scanning microscope equipped with a 10× objective to study the penetration of the particles through the mucus. Z-stacks were acquired once the mucus sample surface was identified. The stained mucus was detected in the red channel (excitation wavelength: 639 nm), whereas the fluorescent LP was visualised in the green channel (excitation wavelength: 488 nm).

### 2.10. Statistical Analysis

All results are presented as mean ± standard deviation (SD). The Kolmogorov–Smirnov test verified the data’s normality, and Spearman’s test verified the heteroscedasticity. Two-way ANOVA test with Dunnett’s multiple comparisons test was used to evaluate differences among groups. *p*-values < 0.05 were regarded as statistically significant. For all analyses, GraphPad Prism 10.0.0 was used (GraphPad Software, San Diego, CA, USA).

## 3. Results and Discussion

### 3.1. Evaluation of Liposomes According to Size

Among the properties that can be modulated, size is a key factor to consider once LPs enter the respiratory system, as it influences their interaction with the immune system, migration across lung barriers, internalisation by cells, and translocation to the bloodstream [40]. In our case, we evaluated fluorescently labelled LPs of three different sizes (100 nm, 200 nm, and 600 nm) to study their ability to penetrate and pass through the APM and be internalised by pulmonary epithelial cells (A549 and Calu-3).

#### 3.1.1. Synthesis and Physicochemical Characterisation of Liposomes

LPs of different sizes were synthesised using the thin-film hydration method, followed by an extrusion process with polycarbonate membranes of different pore sizes (600, 200, and 100 nm). DPPC was used for the synthesis due to its role as a major component of the pulmonary surfactant, which confers increased biocompatibility after administration into the pulmonary system. In addition, DOPE was incorporated because it has been observed to facilitate fusion with cell membranes and LP uptake, favouring drug delivery [41].

**Figure 1:**
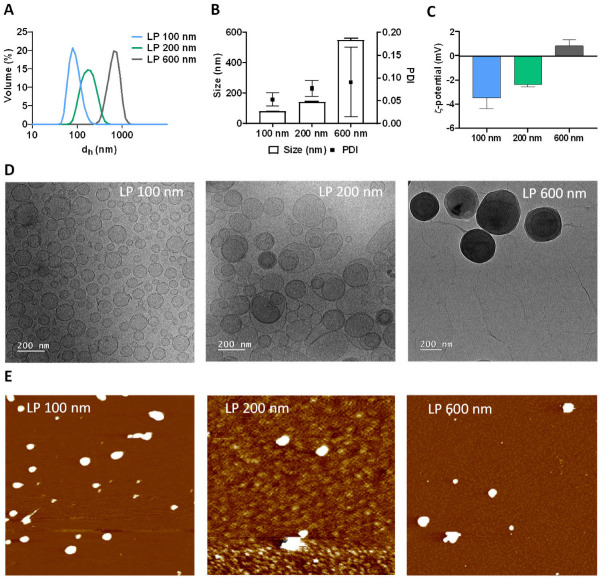
Characterisation of LP 100 nm, LP 200 nm, and LP 600 nm. (**A**) Hydrodynamic diameter. (**B**) Polydispersity index. (**C**) Zeta potential. (**D**) Representative cryo-TEM images and (**E**) AFM images.

Physicochemical characterisation of LPs included size determination, distribution, and stability using different techniques. Through DLS analysis, the measured hydrodynamic diameters obtained were 106.2 ± 0.9 nm, 199 ± 1 nm, and 661 ± 2 nm (Figure 1A). Accordingly, the samples were designated as LP 100 nm, LP 200 nm, and LP 600 nm. The corresponding polydispersity indices (PDI) were 0.05 ± 0.01, 0.07 ± 0.01, and 0.09 ± 0.07 for LP 100 nm, LP 200 nm, and LP 600 nm, indicating relatively homogeneous size distributions and high monodispersity across all formulations, particularly for LP 100 nm [42] (Figure 1B). Zeta potential values (Figure 1C), measured by electrophoretic light scattering, were −3.5 ± 0.9 mV, −2.4 ± 0.2 mV, and 0.8 ± 0.5 mV. These values fall within the range commonly reported for neutral liposomal formulations, indicating an overall neutral surface charge [43]. Therefore, electrostatic effects are unlikely to account for notable differences in colloidal stability between formulations. Any observed differences in dispersion or tendency to aggregate are more plausibly related to size-dependent physical factors rather than to changes in surface charge [42]. In addition, images obtained by cryo-TEM revealed average sizes of 81 nm for the LP 100 nm, 110 nm for the LP 200 nm, and 350 nm for the LP 600 nm (Figure 1D). However, cryo-TEM is commonly used to effectively visualise lipid vesicles ranging from 20 to 400 nm. Vesicles larger than 400 nm may not vitrify properly due to the thickness limitations of the ice layer in cryo-TEM grids, as is visible in the cryo-TEM images of LP 600 nm [44]. Therefore, AFM was also used to characterise the LP size. The obtained diameters were 93.5 nm, 182.6 nm, and 280 nm for LP 100, 200, and 600 nm, respectively (Figure 1E). The differences observed between the techniques, mainly for the LP 600 nm, can be attributed to the inherent limitations of each characterisation method. Cryo-TEM measurements allow visualisation of individual particles under vitrified conditions, thereby minimising aggregation. Nonetheless, the technique may underestimate factors such as morphological changes and vesicle rearrangement, which are more likely to occur in larger liposomes during the freezing and drying processes [45]. Regarding AFM measurements, larger liposomes tended to exhibit denser lipid packing, protecting their hydrophobic regions from contact with the surrounding medium. As a result, van der Waals and hydrophobic interactions with the AFM substrate are weaker, reducing their ability to adhere to the surface and, in some cases, causing them to remain suspended in solution [46]. This could explain the lower agreement in the size values obtained for the LP 600 nm with different measurement techniques. The DLS technique provides a more representative measurement of the real size of the LPs in solution, as it determines the hydrodynamic diameter, which includes the hydration layer and possible interactions with the dispersion medium. This may lead to an overestimation of size compared with microscopy techniques, but it also allows for the assessment of particles in suspension and in dynamic movement, more accurately reflecting their behaviour in biological media [47]. These discrepancies highlight the relevance of combining different techniques to obtain a complete and more accurate characterisation of LPs.

#### 3.1.2. Effect of Liposome Size on Cytotoxicity 

Despite being broadly considered biocompatible and unlikely to trigger significant toxic effects in the body [48], it is crucial to evaluate this aspect to ensure that modifications in their properties do not affect the toxicological profile. To verify whether the change in size could affect their cytotoxic capacity, a cell viability assay was performed with the three types of LPs using the MTT assay.

**Figure 2:**
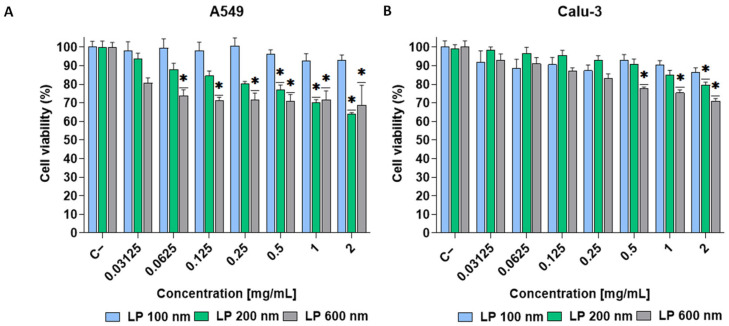
Cell viability (MTT assay) of A549 (**A**) and Calu-3 (**B**) exposed for 24 h to LP 100 nm, LP 200 nm, and LP 600 nm at different concentrations (dilutions 1:2 starting at a concentration of 2 mg/mL). Results are expressed as the mean ± SD of six replicates per tested condition and three independent assays (*n* = 18). Asterisks (*) indicate statistically significant differences compared with the untreated cells (C−) (*p* < 0.05).

A549 and Calu-3 cells were exposed to different concentrations of LPs (0.031–2 mg/mL) for 24 h. In both cell lines, cell viability above 70% was observed for all sizes evaluated, a threshold at which the compound was considered to have no cytotoxic effect [49]. Despite this, for the A549 cell line (Figure 2A), significant differences with respect to the negative control (untreated cells) were observed at concentrations starting from 0.06 mg/mL for LP 600 nm and 0.5 mg/mL for LP 200 nm. In the Calu-3 cell line (Figure 2B), such differences were observed with LP 600 nm from 0.5 mg/mL onwards and only at 2 mg/mL with LP 200 nm. In the case of exposure to LP 100 nm, no significant differences were observed with respect to the negative control at any of the concentrations tested in both cell lines. This tendency towards increased toxicity with increasing size could be explained by the fact that larger LPs have a greater tendency to aggregate and are difficult to internalise by the cells [50,51]. This can lead to their accumulation on the cell surface, mechanical damage, and cellular stress, which trigger adverse cellular responses. It has been reported that increasing the size of liposomes leads to higher levels of proinflammatory cytokines, which can generate an uncontrolled inflammatory response [52].

#### 3.1.3. Size-Dependent Cellular Uptake of LP 

The evaluation of pharmacological compounds targeting the lung has traditionally been performed in cells exposed to submerged conditions. However, this type of exposure does not accurately reflect the conditions that occur in the lungs in vivo, where drugs come into contact with cells through air–liquid exposure [53]. In this study, both LLI and ALI exposure were performed on both cell lines at different time points (0.25, 1, 2, 4, 6, and 16 h). This enabled an evaluation of the differences between both types of exposure, as well as the influence of the presence of pulmonary mucus in Calu-3 cells compared with its absence in A549 cells.

**Figure 3:**
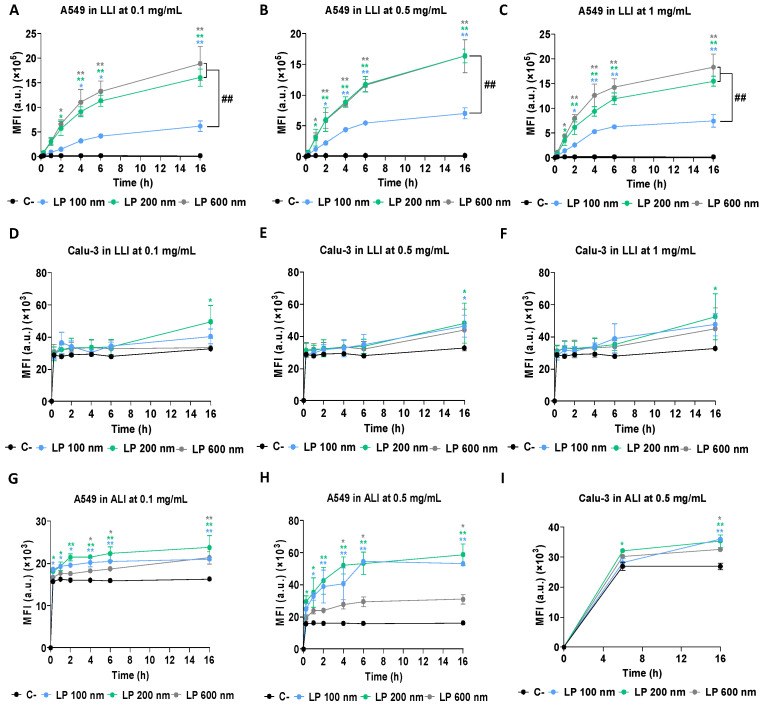
Cellular uptake of LP 100 nm, 200 nm, and 600 nm in A549 and Calu-3 cell lines under different exposure models. (**A**–**C**) Uptake in A549 cells in LLI exposure at 0.1 mg/mL, 0.5 mg/mL, and 1 mg/mL. (**D**–**F**) Uptake by Calu-3 cells exposed to LLI at the same concentrations. (**G**,**H**) Uptake in A549 cells under ALI at 0.1 mg/mL and 0.5 mg/mL. (**I**) Uptake in Calu-3 cells in ALI at 0.5 mg/mL. All exposures were evaluated at 0.25, 1, 2, 4, 6, and 16 h. Results are expressed as the mean ± SD of three replicates per tested condition and three independent assays (*n* = 9). Asterisks (*) indicate statistically significant differences compared with untreated cells (C−), and number signs (#) indicate significant differences within conditions. * *p* < 0.05, ** *p* < 0.01; # *p* < 0.05, ## *p* < 0.01.

For both types of exposure, LPs fluorescently labelled with the hydrophobic dye DiOC18 were quantified by flow cytometry. In the case of LLI exposure with the A549 cell line, at all tested concentrations (0.1, 0.5, and 1 mg/mL), after 4 h of exposure, there was a significant increase in cellular acquisition of LPs (Figure 3A–C). Moreover, at all times evaluated, both LP 600 nm and LP 200 nm showed higher cell acquisition than LP 100 nm. Although this indicates increased cellular uptake, it should be noted that the higher lipid content of larger liposomes could also contribute to the higher signal, given the nature of the lipophilic label DiOC18. In the Calu-3 cell line (Figure 3D–F), significant differences were only observed at 16 h for LP 200 nm at all concentrations and for LP 100 nm at 0.5 mg/mL. For ALI exposure, as mentioned in the methodology section, 1 mg/mL was discarded due to the risk of nebuliser clogging. In the case of A549 cells (Figure 3G,H), LP 100 nm and 200 nm showed significant differences with respect to the negative control at all times tested, while the differences were also significant for LP 600 nm from 4 h of exposure onwards. In addition, at 0.5 mg/mL, a significantly higher acquisition was observed for the LP 100 nm and 200 nm, compared with the LP 600 nm. For the Calu-3 cell line (Figure 3I), results showed that at 0.5 mg/mL and 6 h of exposure, only LP 200 nm showed differences with respect to the negative control, while at 16 h, all sizes showed such differences. In this case, results for the 0.1 mg/mL concentration and the first times evaluated (0.25 h, 1 h, 2 h, and 4 h) were not included, as they were not detected fluorometrically. The impossibility of detecting lower concentrations and shorter times may have been due to the presence of the mucus barrier preventing cellular uptake of LPs. This contrasts with A549 cells, where this barrier is not present, allowing greater interaction and uptake of LPs, even at earlier times and lower concentrations [54]. The differences observed between the LLI and ALI exposure systems can be associated, in part, with the inherent oversizing of the LLI model. In this case, when particles are suspended in the culture medium for prolonged periods, the rate of contact and deposition on the cell surface may increase, facilitating their internalisation [55]. However, under more physiological exposure conditions, such as ALI, where lower deposition efficiency occurs due to the use of the nebulisation system, cell acquisition is limited [56]. These methodological differences underline the importance of adjusting the concentrations according to the characteristics of each exposure system and cell line used. In this regard, when looking at the differences according to the size of the LPs in ALI, LPs 100 nm and 200 nm showed higher cellular uptake than LP 600 nm. This contrast may be attributed to the physical characteristics of the larger liposomes, as particles larger than 500 nm are more easily trapped in the mucosal mesh and are more difficult to internalise by cells, as previously reported [51,57]. In contrast, smaller liposomes seem to overcome these barriers more efficiently.

#### 3.1.4. Interaction with APM as a Function of Liposome Size

When designing nanomaterials for the treatment of lung diseases, it is essential to consider the properties of the mucosal layer that covers and protects the lung epithelial surface. The structure of the mesh is highly heterogeneous, characterised by variable pore sizes and complex viscoelastic properties. This is a crucial factor in the differential penetration of external particles, depending on their surface properties and size [58]. In this study, the behaviour of LPs was analysed as a function of their size when interacting with the synthesised APM. In this context, artificial pulmonary mucus (APM) was employed as a reproducible and well-controlled model to evaluate liposome–mucus interactions under standardised conditions. While native human or animal mucus provides higher physiological relevance, its limited availability, high inter-sample variability, and susceptibility to compositional changes during collection and storage hinder systematic comparisons. The use of APM, therefore, allowed consistent interpretation of liposome diffusion and retention within the mucus layer.

**Figure 4:**
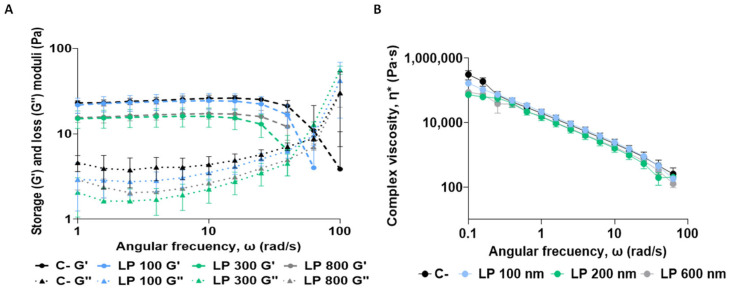
Rheological behaviour of mucus samples using rotational rheometry with plate–plate geometry. The storage (G′) and loss (G″) modulus (**A**) and complex viscosity (η*) (**B**) were analysed in each sample mixing each liposome (100 nm, 200 nm, and 600 nm) with APM. A mucus sample mixed with PBS was used as a negative control (C−). Results are expressed as the mean ± SD of three independent assays (*n* = 3).

The initial analysis focused on evaluating the rheological properties of the APM. Oscillation tests provided information on the viscoelastic behaviour of mucus, evaluating both its storage modulus (G′), which reflects the energy storage capacity, and its loss modulus (G″), which indicates the energy loss (Figure 4A). Based on these parameters, the complex viscosity of the material was calculated to understand its response to oscillatory shear stress. Subsequently, the rheological properties of the APM after interaction with the LP were analysed (Figure 4B). The results showed no significant changes in viscoelastic parameters following LP exposure, suggesting that under the tested conditions, LPs did not substantially alter the structural integrity or mechanical behaviour of the APM. Although the designed APM exhibits mechanical properties that can mimic native pulmonary mucus, it does not fully replicate the dynamic, heterogeneous, and compositionally complex nature of mucus in vivo, which could further influence the interaction with nanomaterials and how these compounds move through the mucus layer [39]. These factors can alter the network structure and, therefore, its rheology. Thus, these parameters must be validated under more representative conditions of the in vivo process, including more complex interactions with the pulmonary mucus.

**Figure 5:**
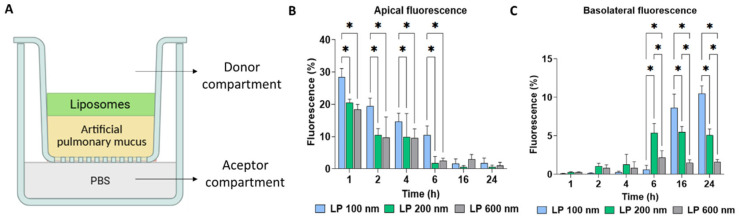
Mucus penetration of LPs of three different sizes (100, 200, and 600 nm) at 1 mg/mL and different time points in APM. Schematic illustration of the in vitro penetration assay across the APM (**A**). Quantification of LP retained in the apical (**B**) and basolateral (**C**) sides was assessed by fluorescence. Values are presented as the average ± standard deviation of three replicates per tested condition and three independent assays (*n* = 9). Asterisks (*) indicate statistically significant differences within conditions (*p* < 0.05). (**A**) was created in https://BioRender.com (accessed on 1 January 2026).

The ability of LPs, as a function of size, to cross the mucus layer was also evaluated. For this assessment, APM was deposited on the apical side of Transwell inserts, and, subsequently, LP samples were added to their surface (Figure 5A). At different time points (1, 2, 4, 6, 16, and 24 h), the percentage of fluorescence present in both the apical (Figure 5B) and basolateral (Figure 5C) sides was collected and analysed. The results showed that while the percentage of fluorescence on the apical side gradually decreased, there was an increase on the basolateral side, indicating permeability of LPs across the mucus, which was more evident after 6 h of exposure. At that time, on the basolateral side, LP 200 nm showed a higher percentage of fluorescence than LP 100 nm and LP 600 nm. However, at 16 h and 24 h, LP 100 nm exhibited a significantly higher basolateral fluorescence than the other formulations. This behaviour may be attributed to differences in transport mechanisms. The intermediate-sized LP 200 nm likely underwent more rapid sedimentation early on, enhancing short-term penetration. In contrast, although LP 600 nm may also be influenced by sedimentation, their larger size likely caused entrapment within the mucus mesh pores, a phenomenon previously reported for nanomaterials interacting with porcine respiratory mucus [59]. At prolonged times (16 h and 24 h), LP 100 nm showed a greater ability to cross the mucus layer and reach the basolateral side than the larger ones. In this case, the penetration of LP 100 nm into the mucus layer depends on their diffusion capacity, which delays their migration at shorter exposure times. Conversely, at longer ones, they benefit from sustained diffusion, allowing gradual accumulation and deeper penetration.

**Figure 6:**
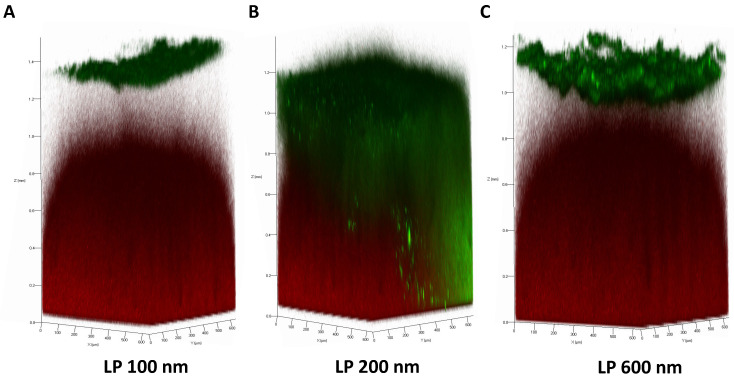
Z-stacks obtained by confocal laser scanning microscopy of LP diffusion (green) in APM (red) after 4 h of incubation: (**A**) LP 100 nm; (**B**) LP 200 nm; (**C**) LP 600 nm.

Images obtained by confocal microscopy followed the same trend as the results obtained for the assay previously described. Images of fluorescently labelled LP (green) deposited on the stained mucosal layer (red) were obtained after 4 h of incubation (Figure 6). As mentioned above, regarding the 6 h exposure time, LP 200 nm showed a greater ability to migrate through the mucus layer compared with the other sizes.

Based on the obtained results, particle size emerges as a critical factor in the design of pulmonary liposomal therapeutics, as it influences the fate of the compound within the lung. Overall, larger liposomes (LP 600 nm) showed reduced colloidal stability, higher cytotoxicity at increasing concentrations, and limited ability to penetrate the pulmonary mucus mesh, which restricts their access to the lung epithelium. In contrast, liposomes smaller than 200 nm, particularly LP 100 nm, exhibited superior colloidal stability, lower cytotoxicity, and enhanced mucus penetration over time. These features allow smaller liposomes to diffuse through the mucus network and reach epithelial cells more efficiently, whereas larger liposomes are prone to mucus entrapment and clearance. Therefore, LP 100 nm emerged as the most suitable formulation for pulmonary delivery among the tested sizes.

### 3.2. Evaluation of Liposomes According to the Presence of PEG

PEGylation has been established as an effective strategy to improve the therapeutic activity of diverse nanomaterials owing to several differential properties, including its enhanced mucopenetrating capacity. However, less characterised critical aspects have also been identified, such as their difficulty in internalisation by cells [60,61].

PEGylation of LP 100 nm was performed (hereafter referred to as LP-PEG) to assess the influence of PEG on the capacity of LPs to be taken up by cells and migrate through pulmonary mucus. In addition, their cytotoxic effects, cellular internalisation capacity, and interaction with APM were compared with those of unmodified LP 100 nm (hereafter referred to as LP). PEG functionalisation of the LP 600 nm was discarded because their unmodified versions already showed the highest levels of cytotoxicity and retention in the mucus, as well as low levels of cellular uptake. Likewise, although LP 200 nm exhibited cellular internalisation efficacy comparable to that of LP 100 nm, the smaller particles showed superior migration through the APM during prolonged exposure, along with reduced cytotoxicity.

#### 3.2.1. Physicochemical Characterisation of Liposomes with PEG

The physicochemical characterisation of LP-PEG was carried out following the same methodology as the initial size evaluation.

**Figure 7:**
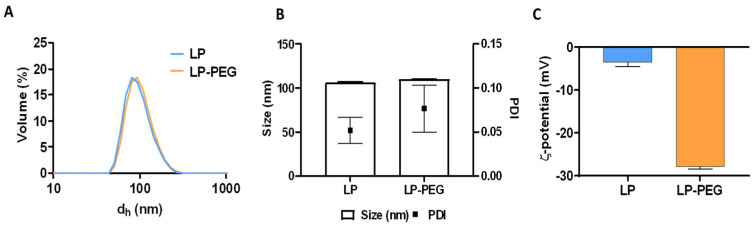
Characterisation of LP and LP-PEG by measurements of hydrodynamic diameter (d_h_) (**A**), polydispersity indices (**B**), and zeta potential (**C**).

DLS measurements (Figure 7A) determined a hydrodynamic diameter of 109.9 ± 0.31 nm for the LP-PEG and a polydispersity index (Figure 7B) of 0.07 ± 0.02, while for the LP, their hydrodynamic diameter was 106.2 ± 0.9 nm and their polydispersity index 0.05 ± 0.01, showing for both types of samples similar sizes and a homogeneous particle distribution, given the measured low polydispersity index (<0.1). This suggests that these were stable formulations without significant aggregation. However, notable differences were observed with respect to the zeta potential (Figure 7C). While for the LP the surface charge was −3.48 ± 0.9 mV, for the LP-PEG it became more negative, being −27.82 ± 0.55 mV. This indicates that the presence of DSPE-PEG in LP-PEG, which is negatively charged due to the ionisation of its phosphate group in the DSPE moiety under physiological conditions, confers a higher colloidal stability. Consequently, its association with other liposomes is avoided, which may result in the suppression of complement activation and recognition by the phagocytic system, thereby increasing its biodistribution and circulation time [62].

As for the TEM and AFM measurements, similar parameters were reported for both types of compositions.

#### 3.2.2. Effect of Liposome PEGylation on Cytotoxicity

**Figure 8:**
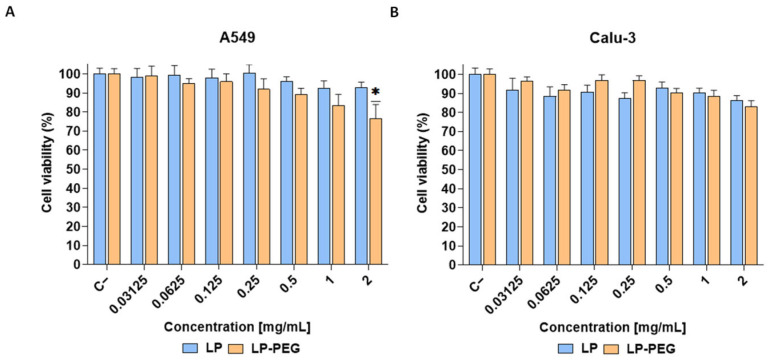
Cell viability (MTT assay) of A549 (**A**) and Calu-3 (**B**) cells exposed for 24 h to LP and LP-PEG at different concentrations (dilutions 1:2 starting at a concentration of 2 mg/mL). Results are expressed as means ± SD of six replicates per tested condition and three independent assays (*n* = 18). Asterisks (*) indicate significant differences with respect to the untreated cells (C−) (*p* < 0.05).

PEG has been widely used for the formulation of cosmetic and pharmaceutical ingredients due to its ability to increase solubility, lower immunogenicity, and increase stability [63,64,65]. In this study, the cytotoxic capacity of LP-PEG was evaluated in comparison with LP by performing an MTT assay under the same conditions as for the previous size-dependent cytotoxicity assessment (Figure 8). The results did not show a cytotoxic effect for the tested concentrations in either cell line. Viability > 80% was observed in all conditions, except for LP-PEG at the highest concentration (2 mg/mL) in the A549 cell line, which showed a significant decrease in cell viability compared with the negative control (untreated cells). These findings highlight the need to perform a thorough toxicological evaluation for each synthesised formulation, since diverse parameters, such as functionalisation and composition, in addition to size, can influence its toxicity.

#### 3.2.3. PEGylation-Dependent Cellular Uptake of Liposomes

**Figure 9:**
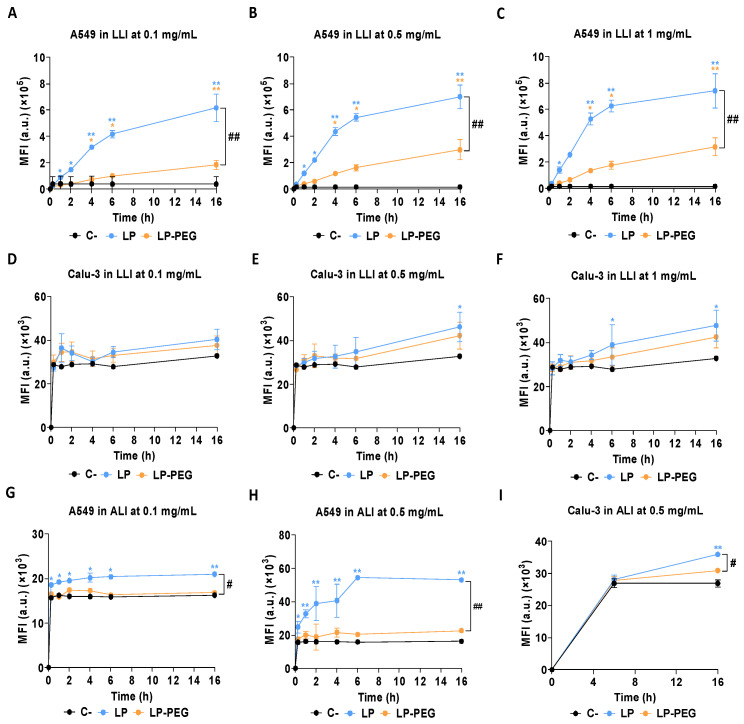
Cellular uptake of LP and LP-PEG in A549 and Calu-3 cell lines in different exposure models. (**A**–**C**) Uptake in A549 cells in LLI exposure at 0.1 mg/mL, 0.5 mg/mL, and 1 mg/mL. (**D**–**F**) Uptake by Calu-3 cells exposed to LLI at the same concentrations. (**G**,**H**) Uptake in A549 cells in ALI exposure at 0.1 mg/mL and 0.5 mg/mL. (**I**) Uptake in Calu-3 cells in ALI at 0.5 mg/mL and two time points (6 h and 16 h). All exposures were evaluated at 0.25, 1, 2, 4, 6, and 16 h unless otherwise stated. Results are expressed as means ± SD of three replicates per tested condition and three independent assays (*n* = 9). Asterisks (*) indicate statistically significant differences compared with untreated cells (C−), and number signs (#) indicate significant differences within conditions. * *p* < 0.05, ** *p* < 0.01; # *p* < 0.05, ## *p* < 0.01.

To assess the cellular uptake of LP and LP-PEG, the same procedure as that used for the size effect evaluation was followed. Although no significant differences were detected in the Calu-3 cell line under LLI exposure (Figure 9D–F), results obtained at all tested concentrations in LLI with the A549 cell line (Figure 9A–C) and ALI with both cell lines (Figure 9G–I) exhibited a significantly higher acquisition of LP compared with LP-PEG. This behaviour can be attributed to the hydrophilic nature of PEG, which forms a hydrated steric layer around the liposomes, reducing their adhesion to the cell membrane and limiting subsequent internalisation, as previously reported [66,67]. In this sense, PEGylation can be advantageous for evading immune recognition, reducing uptake by macrophages, and prolonging liposome residence time in the lung [68]. However, it may be detrimental when rapid and efficient cellular internalisation is required, such as in the delivery of genetic material or therapies targeting intracellular or tumour cells [69].

#### 3.2.4. Interaction with APM as a Function of PEGylation 

Similar to our initial evaluation of size-dependent migration of liposomes, functionalised particles were analysed in rheology studies, migration assays through APM, and confocal microscopy.

**Figure 10:**
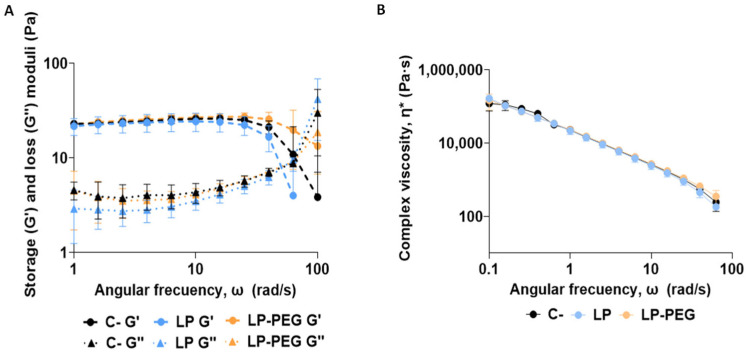
Rheological behaviour of mucus samples using rotational rheometry with plate geometry. The elastic/storage (G′), viscous/loss (G″) modulus (**A**), and complex viscosity (η*) (**B**) were analysed in each sample mixing each liposome (LP and LP-PEG) with APM. APM mixed with PBS was used as a negative control (C−). Results are expressed as means ± SD of three independent assays (*n* = 3).

The rheological results showed no change in the rheological properties of mucus upon interaction with PEG (Figure 10). As previously stated, studies with more complex models are essential to confirm whether real rheological changes occur upon the interaction of the polymer with the mucous membrane.

**Figure 11:**
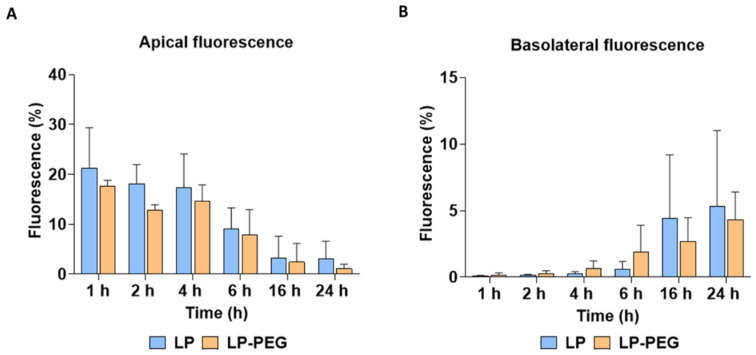
Mucus penetration of LP and LP-PEG at 1 mg/mL and different time points (1 h, 2 h, 4 h, 6 h, 16 h, and 24 h) in APM. Quantification of liposomes retained in the apical (**A**) and basolateral (**B**) compartments was assessed using fluorescence. Values are presented as average ± standard deviation of three replicates per tested condition and three independent assays (*n* = 9).

Regarding the penetration ability of PEG across APM, previous studies have shown that different types of nanomaterials, when functionalised with PEG, increase their mucopenetrating capacity compared with their counterparts without PEG [57,70,71]. However, the migration assay did not reflect this difference, which could be attributed to limitations in the experimental conditions, such as the sensitivity of the assay for detecting slight differences in penetration (Figure 11).

**Figure 12:**
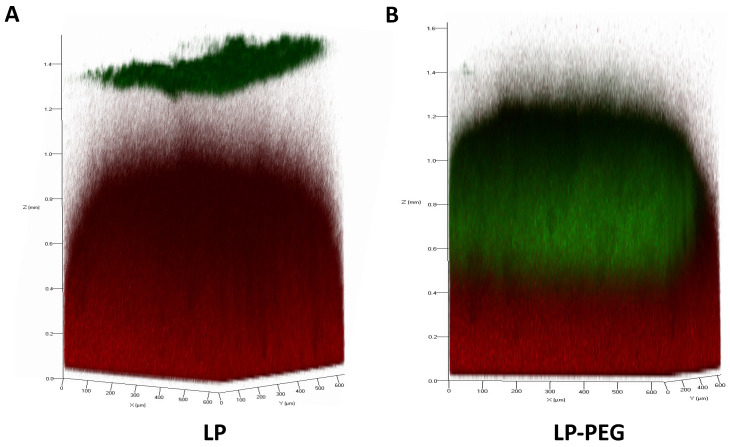
Z-stacks obtained by confocal laser scanning microscopy of LP diffusion (green) in APM (red) after 4 h of incubation: (**A**) LP; (**B**) LP-PEG.

According to the results obtained by confocal microscopy (Figure 12), the images obtained followed the same guidelines as those carried out based on size; the images of the green-labelled liposomes deposited on the red-labelled APM were taken after 4 h of incubation. The results revealed that LP-PEG exhibited enhanced migratory capacity compared with LP. This observation aligns with previously reported findings in the literature, as discussed earlier.

Depending on the site of action of the administered drug, functionalisation of LP with PEG should be considered. In strategies where particles are not intended to act as intracellular delivery systems, PEGylation facilitates rapid migration to the epithelial surface and enables extracellular activity without the risk of cellular internalisation. This is particularly advantageous, as it reduces the likelihood of elimination via mucociliary clearance or uptake by alveolar macrophages.

## 4. Conclusions

This study highlights the critical importance of evaluating the influence of liposome size and PEGylation when designing formulations for the treatment of lung diseases. By addressing the influence of these parameters, this study provides a comprehensive framework aimed at optimising the therapeutic efficacy of liposomes while minimising potential adverse effects.

A thorough and effective assessment of the applicability of liposomes as pulmonary drug delivery systems requires detailed characterisation using a variety of techniques to overcome methodological limitations and variability. While LLI exposure has traditionally been used, ALI exposure allows for a more physiologically relevant model of the actual conditions encountered in the respiratory tract. Likewise, the selection of an appropriate cell line is crucial, as it enables a global approach to the behaviour of liposomes through the lung epithelium and the different barriers they can encounter across each region. Although the use of in vitro lung mucus models allows the study of the interaction with the formulation of interest, these systems do not fully replicate the complexity and dynamic properties of real pulmonary mucus in vivo. Therefore, complementary studies are necessary to bridge this gap and provide a more accurate representation of physiological conditions.

The size of liposomes plays a pivotal role in determining their colloidal stability, interaction with the mucosal layer, and cellular internalisation. As the particle size increases, their stability in suspension decreases, leading to a higher tendency for aggregation. Consequently, optimal stability values were obtained for LP 100 nm. Furthermore, LP 100 nm showed lower cytotoxicity and greater migration capacity through mucus over prolonged periods, even though similar cellular internalisation values were obtained for LP 200 nm.

PEGylation also has a significant impact on liposome performance. The addition of DSPE-PEG to LP 100 nm improved colloidal stability compared with liposomes without modification. In addition, PEGylation was associated with reduced cellular internalisation but enhanced migration through the mucosal layer. This distinction underscores the importance of tailoring liposome properties to align with specific therapeutic objectives, balancing internalisation into cells with mucus penetration. For instance, PEGylated liposomes may be better suited for applications requiring retention on the epithelial surface outside of the cells, whereas non-PEGylated liposomes might be more effective for therapies targeting the intracellular delivery of active compounds.

In conclusion, understanding the influence of size and PEGylation is crucial for evaluating liposome interactions with cellular and non-cellular biological barriers and for designing formulations with properties optimised for specific therapeutic targets. Furthermore, the use of complementary characterisation techniques and the selection of appropriate experimental and exposure models are fundamental to ensure their effectiveness.

## Data Availability

Data is contained within the article.

## References

[1] Levine S., Marciniuk D., Aglan A., Celedón J.C., Fong K., Horsburgh R., Malhotra A., Masekela R., Mortimer K., Redde H., Writing Committee (2021). The Global Impact of Respiratory Disease Third Edition.

[2] Jin Z., Gao Q., Wu K., Ouyang J., Guo W., Liang X.J. (2023). Harnessing inhaled nanoparticles to overcome the pulmonary barrier for respiratory disease therapy. Adv. Drug Deliv. Rev..

[3] Wang B., Wang L., Yang Q., Zhang Y., Qinglai T., Yang X., Xiao Z., Lei L., Li S. (2024). Pulmonary inhalation for disease treatment: Basic research and clinical translations. Mater. Today Bio.

[4] Darquenne C., Fleming J.S., Katz I., Martin A.R., Schroeter J., Usmani O.S., Venegas J., Schmid O. (2016). Bridging the Gap Between Science and Clinical Efficacy: Physiology, Imaging, and Modeling of Aerosols in the Lung. J. Aerosol Med. Pulm. Drug Deliv..

[5] Kuzmov A., Minko T. (2015). Nanotechnology approaches for inhalation treatment of lung diseases. J. Control. Release.

[6] He S., Gui J., Xiong K., Chen M., Gao H., Fu Y. (2022). A roadmap to pulmonary delivery strategies for the treatment of infectious lung diseases. J. Nanobiotechnology.

[7] Yue L., Zhang X., Zhao C., Chen R., Chen X., Rao L. (2023). Inhaled drug delivery: Past, present, and future. Nano Today.

[8] Button B., Cai L.H., Ehre C., Kesimer M., Hill D.B., Sheehan J.K., Boucher R.C., Rubinstein M. (2012). Periciliary Brush Promotes the Lung Health by Separating the Mucus Layer from Airway Epithelia. Science.

[9] Alp G., Aydogan N. (2020). Lipid-based mucus penetrating nanoparticles and their biophysical interactions with pulmonary mucus layer. Eur. J. Pharm. Biopharm..

[10] Sanders N., Rudolph C., Braeckmans K., De Smedt S.C., Demeester J. (2009). Extracellular barriers in respiratory gene therapy. Adv. Drug Deliv. Rev..

[11] Boucher R.C. (2007). Airway surface dehydration in cystic fibrosis: Pathogenesis and therapy. Annu. Rev. Med..

[12] Ramos F.L., Krahnke J.S., Kim V. (2014). Clinical issues of mucus accumulation in COPD. Int. J. Chronic Obstr. Pulm. Dis..

[13] Shaikh M.A.J., Goyal K., Afzal M., Roopashree R., Kumari M., Krithiga T., Panigrahi R., Saini S., Ali H., Imran M. (2025). Liposome-encapsulated therapies: Precision medicine for inflammatory lung disorders. Nano TransMed.

[14] Mehta P.P., Ghoshal D., Pawar A.P., Kadam S.S., Dhapte-Pawar V.S. (2020). Recent advances in inhalable liposomes for treatment of pulmonary diseases: Concept to clinical stance. J. Drug Deliv. Sci. Technol..

[15] Hua S., Wu S.Y. (2013). The use of lipid-based nanocarriers for targeted pain therapies. Front. Pharmacol..

[16] Van Rijt S.H., Bein T., Meiners S. (2014). Medical nanoparticles for next generation drug delivery to the lungs. Eur. Respir. J..

[17] Panthi V.K., Fairfull-Smith K.E., Islam N. (2024). Antibiotic loaded inhalable liposomal nanoparticles against lower respiratory tract infections: Challenges, recent advances, and future perspectives. J. Drug Deliv. Sci. Technol..

[18] Feng X., Shi Y., Zhang Y., Lei F., Ren R., Tang X. (2024). Opportunities and Challenges for Inhalable Nanomedicine Formulations in Respiratory Diseases: A Review. Int. J. Nanomed..

[19] Ponkshe P., Feng S., Tan C. (2021). Inhalable liposomes for treating lung diseases: Clinical development and challenges. Biomed. Mater..

[20] Liu Q., Guan J., Qin L., Zhang X., Mao S. (2020). Physicochemical properties affecting the fate of nanoparticles in pulmonary drug delivery. Drug Discov. Today.

[21] Jaradat E., Meziane A., Lamprou D.A. (2024). Conventional vs PEGylated loaded liposomal formulations by microfluidics for delivering hydrophilic chemotherapy. Int. J. Pharm..

[22] Sercombe L., Veerati T., Moheimani F., Wu S.Y., Sood A.K., Hua S. (2015). Advances and challenges of liposome assisted drug delivery. Front. Pharmacol..

[23] Maja L., Željko K., Mateja P. (2020). Sustainable technologies for liposome preparation. J. Supercrit. Fluids.

[24] Leong E.W.X., Ge R. (2022). Lipid Nanoparticles as Delivery Vehicles for Inhaled Therapeutics. Biomedicines.

[25] Xu L., Wang X., Liu Y., Yang G., Falconer R.J., Zhao C.X. (2022). Lipid Nanoparticles for Drug Delivery. Adv. NanoBiomed Res..

[26] Costabile G., Conte G., Brusco S., Savadi P., Miro A., Quaglia F., d’Angelo I., Ungaro F. (2024). State-of-the-Art Review on Inhalable Lipid and Polymer Nanocarriers: Design and Development Perspectives. Pharmaceutics.

[27] van der Worp H.B., Howells D.W., Sena E.S., Porritt M.J., Rewell S., O’Collins V., Macleod M.R. (2010). Can Animal Models of Disease Reliably Inform Human Studies?. PLoS ONE.

[28] Allouche Y., Marchetti S., Bengalli R., Motta G., Pagliarulo L., Cazier F., Achard S., Fadel M., Mantecca P., Courcot D. (2025). Comparison of submerged and air liquid interface exposure: Limitations and differences in the toxicological effects evaluated in bronchial epithelial cells. Environ. Res..

[29] de Souza Carvalho C., Daum N., Lehr C.M. (2014). Carrier interactions with the biological barriers of the lung: Advanced in vitro models and challenges for pulmonary drug delivery. Adv. Drug Deliv. Rev..

[30] Artzy-Schnirman A., Arber Raviv S., Doppelt Flikshtain O., Shklover J., Korin N., Gross A., Mizrahi B., Schroeder A., Sznitman J. (2021). Advanced human-relevant in vitro pulmonary platforms for respiratory therapeutics. Adv. Drug Deliv. Rev..

[31] Doryab A., Tas S., Taskin M.B., Yang L., Hilgendorff A., Groll J., Wagner D.E., Schmid O. (2019). Evolution of Bioengineered Lung Models: Recent Advances and Challenges in Tissue Mimicry for Studying the Role of Mechanical Forces in Cell Biology. Adv. Funct. Mater..

[32] Braakhuis H.M., Gremmer E.R., Bannuscher A., Drasler B., Keshavan S., Rothen-Rutishauser B., Birk B., Verlohner A., Landsiedel R., Meldrum K. (2023). Transferability and reproducibility of exposed air-liquid interface co-culture lung models. NanoImpact.

[33] Lenz A.G., Stoeger T., Cei D., Schmidmeir M., Semren N., Burgstaller G., Lentner B., Eickelberg O., Meiners S., Schmid O. (2014). Efficient bioactive delivery of aerosolized drugs to human pulmonary epithelial cells cultured in air-liquid interface conditions. Am. J. Respir. Cell Mol. Biol..

[34] Frisch S., Boese A., Huck B., Horstmann J.C., Ho D.K., Schwarzkopf K., Murgia X., Loretz B., De Souza Carvalho-Wodarz C., Lehr C.M. (2021). A pulmonary mucus surrogate for investigating antibiotic permeation and activity against Pseudomonas aeruginosa biofilms. J. Antimicrob. Chemother..

[35] Lock J.Y., Carlson T.L., Carrier R.L. (2018). Mucus models to evaluate the diffusion of drugs and particles. Adv. Drug Deliv. Rev..

[36] Zhang H. (2017). Thin-film hydration followed by extrusion method for liposome preparation. Methods Mol. Biol..

[37] Rouser G., Fleischer S., Yamamoto A. (1970). Two dimensional thin layer chromatographic separation of polar lipids and determination of phospholipids by phosphorus analysis of spots. Lipids.

[38] Takechi-Haraya Y., Sakai-Kato K., Abe Y., Kawanishi T., Okuda H., Goda Y. (2016). Observation of liposomes of differing lipid composition in aqueous medium by means of atomic force microscopy. Microscopy.

[39] Huck B.C., Hartwig O., Biehl A., Schwarzkopf K., Wagner C., Loretz B., Murgia X., Lehr C.M. (2019). Macro- And Microrheological Properties of Mucus Surrogates in Comparison to Native Intestinal and Pulmonary Mucus. Biomacromolecules.

[40] Yanagihara S., Kitayama Y., Yuba E., Harada A. (2023). Preparing Size-Controlled Liposomes Modified with Polysaccharide Derivatives for pH-Responsive Drug Delivery Applications. Life.

[41] Mateos-Maroto A., Gai M., Brückner M., da Costa Marques R., Harley I., Simon J., Mailänder V., Morsbach S., Landfester K. (2023). Systematic modulation of the lipid composition enables the tuning of liposome cellular uptake. Acta Biomater..

[42] Danaei M., Dehghankhold M., Ataei S., Hasanzadeh Davarani F., Javanmard R., Dokhani A., Khorasani S., Mozafari M.R. (2018). Impact of particle size and polydispersity index on the clinical applications of lipidic nanocarrier systems. Pharmaceutics.

[43] Clogston J.D., Patri A.K. (2011). Zeta Potential Measurement. Methods Mol. Biol..

[44] Rey-Cadilhac F., Rachenne F., Marquant A., Kee Him J.L., Ancelin A., Foisor V., Morille M., Lyonnais S., Cazevieille C., Missé D. (2025). Characterization of size distribution and markers for mosquito extracellular vesicles. Front. Cell Dev. Biol..

[45] Kuntsche J., Horst J.C., Bunjes H. (2011). Cryogenic transmission electron microscopy (cryo-TEM) for studying the morphology of colloidal drug delivery systems. Int. J. Pharm..

[46] Takechi-Haraya Y., Goda Y., Sakai-Kato K. (2018). Imaging and size measurement of nanoparticles in aqueous medium by use of atomic force microscopy. Anal. Bioanal. Chem..

[47] Maguire C.M., Rösslein M., Wick P., Prina-Mello A. (2018). Characterisation of particles in solution–a perspective on light scattering and comparative technologies. Sci. Technol. Adv. Mater..

[48] Inglut C.T., Sorrin A.J., Kuruppu T., Vig S., Cicalo J., Ahmad H., Huang H.C. (2020). Immunological and toxicological considerations for the design of liposomes. Nanomaterials.

[49] (2009). Biological Evaluation of Medical Devices—Part 5: Tests for In Vitro Cytotoxicity.

[50] Narenji M., Talaee M.R., Moghimi H.R. (2016). Investigating the effects of size, charge, viscosity and bilayer flexibility on liposomal delivery under convective flow. Int. J. Pharm..

[51] Foster K.A., Yazdanian M., Audus K.L. (2001). Microparticulate uptake mechanisms of in-vitro cell culture models of the respiratory epithelium. J. Pharm. Pharmacol..

[52] Yamamoto S., Ishida T., Inoue A., Mikami J., Muraguchi M., Ohmoto Y., Kiwada H. (2002). HEPC-based liposomes trigger cytokine release from peripheral blood cells: Effects of liposomal size, dose and lipid composition. Int. J. Pharm..

[53] Panas A., Comouth A., Saathoff H., Leisner T., Al-Rawi M., Simon M., Seemann G., Dössel O., Mülhopt S., Paur H.R. (2014). Silica nanoparticles are less toxic to human lung cells when deposited at the air-liquid interface compared to conventional submerged exposure. Beilstein J. Nanotechnol..

[54] Meindl C., Öhlinger K., Zrim V., Steinkogler T., Fröhlich E. (2021). Screening for effects of inhaled nanoparticles in cell culture models for prolonged exposure. Nanomaterials.

[55] Raemy D.O., Grass R.N., Stark W.J., Schumacher C.M., Clift M.J.D., Gehr P., Rothen-Rutishauser B. (2012). Effects of flame made zinc oxide particles in human lung cells—A comparison of aerosol and suspension exposures. Part. Fibre Toxicol..

[56] Secondo L.E., Liu N.J., Lewinski N.A. (2017). Methodological considerations when conducting in vitro, air–liquid interface exposures to engineered nanoparticle aerosols. Crit. Rev. Toxicol..

[57] Schuster B.S., Suk J.S., Woodworth G.F., Hanes J. (2013). Nanoparticle diffusion in respiratory mucus from humans without lung disease. Biomaterials.

[58] Yan X., Sha X. (2023). Nanoparticle-Mediated Strategies for Enhanced Drug Penetration and Retention in the Airway Mucosa. Pharmaceutics.

[59] Murgia X., Pawelzyk P., Schaefer U.F., Wagner C., Willenbacher N., Lehr C.M. (2016). Size-Limited Penetration of Nanoparticles into Porcine Respiratory Mucus after Aerosol Deposition. Biomacromolecules.

[60] Hatakeyama H., Akita H., Harashima H. (2013). The polyethyleneglycol dilemma: Advantage and disadvantage of PEGylation of liposomes for systemic genes and nucleic acids delivery to tumors. Biol. Pharm. Bull..

[61] Vila A., Gill H., McCallion O., Alonso M.J. (2004). Transport of PLA-PEG particles across the nasal mucosa: Effect of particle size and PEG coating density. J. Control. Release.

[62] Abe K., Higashi K., Watabe K., Kobayashi A., Limwikrant W., Yamamoto K., Moribe K. (2015). Effects of the PEG molecular weight of a PEG-lipid and cholesterol on PEG chain flexibility on liposome surfaces. Colloids Surf. A Physicochem. Eng. Asp..

[63] Le Khanh H.P., Nemes D., Rusznyák Á., Ujhelyi Z., Fehér P., Fenyvesi F., Váradi J., Vecsernyés M., Bácskay I. (2022). Comparative Investigation of Cellular Effects of Polyethylene Glycol (PEG) Derivatives. Polymers.

[64] Wu L., Chen J., Wu Y., Zhang B., Cai X., Zhang Z., Wang Y., Si L., Xu H., Zheng Y. (2017). Precise and combinatorial PEGylation generates a low-immunogenic and stable form of human growth hormone. J. Control. Release.

[65] Biswas S., Dodwadkar N.S., Deshpande P.P., Torchilin V.P. (2012). Liposomes loaded with paclitaxel and modified with novel triphenylphosphonium-PEG-PE conjugate possess low toxicity, target mitochondria and demonstrate enhanced antitumor effects in vitro and in vivo. J. Control. Release.

[66] Lee J.S., Hwang S.Y., Lee E.K. (2015). Imaging-based analysis of liposome internalization to macrophage cells: Effects of liposome size and surface modification with PEG moiety. Colloids Surf. B Biointerfaces.

[67] Nunes S.S., Silva J.d.O., Fernandes R.S., Miranda S.E.M., Leite E.A., de Farias M.A., Portugal R.V., Cassali G.D., Townsend D.M., Oliveira M.C. (2022). PEGylated versus Non-PEGylated pH-Sensitive Liposomes: New Insights from a Comparative Antitumor Activity Study. Pharmaceutics.

[68] Piñol-Cancer M., Fernández-Méndez L., Carrillo-Romero J., Urkola-Arsuaga A., Azkargorta M., Elortza F., Goñi-De-Cerio F., García-Mouton C., de Alejo C.M.-P., Ismalaj E. (2025). The role of PEGylation in the pulmonary delivery of antifibrotic liposomal therapies. J. Control. Release.

[69] Verhoef J.J.F., Anchordoquy T.J. (2013). Questioning the use of PEGylation for drug delivery. Drug Deliv. Transl. Res..

[70] Lai S.K., Wang Y.Y., Hanes J. (2009). Mucus-penetrating nanoparticles for drug and gene delivery to mucosal tissues. Adv. Drug Deliv. Rev..

[71] Suk J.S., Lai S.K., Boylan N.J., Dawson M.R., Boyle M.P., Hanes J. (2011). Rapid transport of muco-inert nanoparticles in cystic fibrosis sputum treated with N-acetyl cysteine. Nanomedicine.

